# BSAVA Manual of Practical Veterinary Welfare

**DOI:** 10.1017/awf.2023.22

**Published:** 2023-03-16

**Authors:** Chris Laurence

**Affiliations:** Chippenham, UK

This new manual joins the series of very well-respected manuals on a huge range of subjects from BSAVA. The manuals form the basis of practical issues in many veterinary practices so it is good to see one specifically on welfare.

The first chapter from James Yeates is a masterly introduction to the concepts of ethics, welfare and the accompanying methodologies of the Five Freedoms and now the Five Domains. As to be expected from James it takes a logical thought process through what vets and nurses ought to consider as they go about their daily tasks.

The second chapter introduces the concept of ethograms and gives examples of different published and validated recording systems. It then introduces the concept of nursing models and processes referring to the sister BSAVA textbook and moves on to individual care plans. I’m sure these are processes many nurses carry out subconsciously but it is helpful to have them set out. A discussion of enrichment and euthanasia follows and the chapter culminates in some excellent examples of assessment forms.

Chapter 3 sets out the basics of animal behaviour science. A very clear exposition of how animals are motivated and learn includes a number of practical examples from a variety of species. The reader may question why such a long discourse (thirty pages) is necessary in a manual on practical welfare but an understanding of the basic science of behaviour is so important that I think it is a well justified inclusion and it is a very readable explanation. Surely without an understanding of pure science any attempt at practice is doomed to fail?

Next there is a chapter on enrichment that covers a wide range of species. There is a good explanation of why enrichment is so important to captive animal welfare. There are some great examples for the more frequently seen species and particularly for smaller commonly kept mammals where the real-world deficit is probably greatest. The chapter closes with a brief mention of rescue animals whose previous experiences and environment will affect their outlook on enrichment and especially when those animals have been imported from the streets of other countries. It is a shame that only the US-based Fear Free scheme is mentioned as opposed to the UK-based schemes such as Cat Friendly Clinic and Dog Friendly Clinic.

The chapter on nutrition starts with the scientific background and includes a brief discussion of food types including raw food diets. Then follows some tables of food types for herbivores showing what is suitable for which species. That forms an excellent quick reference guide that will be essential reading for practitioners. There follows a section on nutritional disease that inevitably starts with obesity and highlights the incidence and consequences. Some expansion of the section detailing potential action plans for obese patients would have been helpful. The effect of habituation through food of wild species is discussed at some length setting out the consequences of provision of food by humans ranging from violent encounters with animals seeking food, eg bears, to the inevitable road traffic collision consequences for urban foxes.

Chapter 6 provides some excellent examples of training techniques for a wide variety of species and links well with the earlier chapter on the theory of learning and training. Some of the practical examples of training programmes, such as muzzle training, could easily be translated into practice leaflets for clients to help them achieve the desired end result. The examples are perhaps a little heavy on zoo species that most practitioners will never encounter, but they provide a fascinating insight into modern zoo management.

The next chapter introduces the concept of the ‘welfare bank account’ and uses it to illustrate how practices can build the bank balance. Some common sense examples of allowing animals, particularly juveniles, to habituate to car journeys, restraint harnesses and carriers follow. The chapter moves on to discuss management of appointments, separation of species (especially prey from predators) and the layout of consulting rooms, eg allowing cats raised shelves upon which to perch and the use of appropriate pheromones. The authors go on to discuss puppy consultations and then hospitalisation at some length. The importance of low stress handling is emphasised together with discussion of trigger stacking and the excellent ladder of aggression. There follows a section on clinical and surgical procedures highlighting the effects of stress on the process from pre-surgery, surgical technique through to recovery and hospitalisation. This chapter probably has more realistic practical advice than much of the rest of the manual.

Chapter 8 deals with the difficult subject of end-of-life care. It starts with the decision-making process that leads to euthanasia and emphasises that quality of life (QoL) is the most important factor to consider. A simple evaluation tool for owners to complete to illustrate how QoL is, or is not, deteriorating is provided that will help practitioners to lead clients to make the best decision for their pet. It goes on to discuss the bereavement process to help practitioners understand client reactions and indicates where support can be found, particularly for clients with physical and mental health issues. They remind us that post-euthanasia gasping and twitching is the norm for us but not for clients so preparing them is important. The authors also remind us that animals bonded to the euthanased animal may also need our care and advice. The chapter concludes with a very emotive client impact statement that should remind us that pets are ‘not just an animal.’

The penultimate chapter deals with the concept of One Health. There is a wide-ranging discussion of zoonoses, the companion animal-human bond, obesity and antimicrobial resistance. The use of animals for animal-assisted interventions is highlighted with the challenges when aged, infirm or immunosuppressed people are concerned. There is no doubt that our relationship with animals and the environment is critical to our futures.

The final chapter deals with the mental health and welfare of the practice team. Undoubtedly this is one of the major issues in practice today and there is much advice on management techniques and relationships within practice. Clearly, the fourteen pages can only cover a superficial exposition but the further reading and reference list is extensive.

In summary, this is an excellent manual for all those in practice. For the new graduate or just-qualified nurse it should be compulsory reading as there is so much good basic practical advice. And, for those already established in practice, it is a really useful reminder of those things that got forgotten in the daily melee of veterinary work.

